# Osteopathic Physical Exam Findings in Chronic Hepatitis C: A Case Study

**DOI:** 10.7759/cureus.3939

**Published:** 2019-01-22

**Authors:** Justin Chin, Munib Francis, Julie M Lavalliere, Christine M Lomiguen

**Affiliations:** 1 Family Medicine, Touro College of Osteopathic Medicine, New York, USA; 2 Pathology, Touro College of Osteopathic Medicine, New York, USA

**Keywords:** hepatitis c, osteopathic physical exam, osteopathic medicine, liver pathology, hepatic pathology, viscerosomatic reflex, viscerosomatics, chapman's points, chronic hepatitis c, liver dysfunction

## Abstract

Greater than two-thirds of all Hepatitis C virus (HCV) infections are chronic, leading to extrahepatic changes in the body. The objective of this article was to describe various musculoskeletal findings that can be elicited during an osteopathic structural exam in the setting of liver dysfunction along with an overview of hepatic viscerosomatics and Chapman’s points. Osteopathic medicine is a branch of modern medicine that utilizes hands-on diagnosis and treatment through musculoskeletal manifestations in addition to the standard allopathic practices. Herein, we presented a case of untreated chronic HCV infection, complicated by polysubstance abuse, with positive findings on osteopathic physical exam consistent with hepatic pathology.

## Introduction

Hepatitis is an inflammatory disease of the liver, classically involving an initial insult with subsequent injury and immune reaction [1]. The precise mechanism of injury depends on the cause. The most common form of hepatitis worldwide is viral, with around 600 million people affected [2]. Diagnosis is made with serological or antibody studies, while chronic infections require a liver biopsy to evaluate the extent of inflammation and fibrosis [3]. Transformation of acute viral hepatitis to chronic viral hepatitis depends on the specific strain, with 75% to 85% of Hepatitis C virus (HCV) infections becoming chronic [4]. Current noninvasive monitoring of chronic hepatitis C patients is dependent on routine laboratory testing, ultrasound, endoscopic surveillance, and clinical evaluation in patients with cirrhosis; however, traditional clinical examination is geared towards the recognition of hepatic sequelae such as ascites, palmar erythema, and spider angiomas to mention a few [5]. Osteopathic physicians are trained to integrate the medical history of a patient with a palpatory examination through osteopathic manipulative medicine (OMM) that allows the expansion of differential diagnoses and the consideration of somatic dysfunction [6]. Herein, we have described a case of chronic HCV infection, with various musculoskeletal findings that were revealed on osteopathic physical exam.

## Case presentation

A 37-year-old male with a past medical history of attention-deficit hyperactivity disorder, anxiety disorder, untreated Hepatitis C, and history of polysubstance abuse including intravenous (IV) drug use (cocaine, marijuana, and benzodiazepines) presented to the ED requesting a dose of Clonazepam as he had “run out”. Of note, he is frequently seen in the ED for substance-related complaints, most recently two weeks prior. At the time of presentation, he reported that his refill for Clonazepam was not ready and had resorted to using cocaine as a replacement. Upon questioning, he became agitated, walking around the unit with his fists in the air, looking repeatedly at the ceiling and stating “Don’t let them attack”, ultimately requiring four-point restraints and intramuscular Diphenhydramine/Haloperidol/Lorazepam (50 mg/5 mg/2 mg, B52 protocol). 

**Figure 1:**
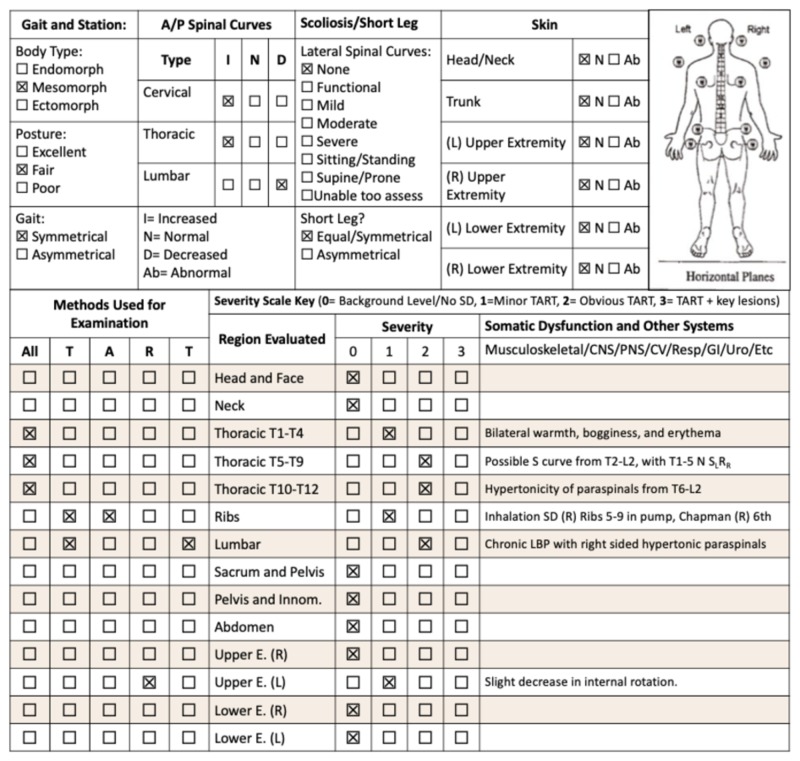
Complete osteopathic physical exam with significant findings Osteopathic examination checklist adapted from the Journal of the American Osteopathic Association

During observation, he was noted to have T wave inversions on telemetry, which were not recorded on subsequent EKG. Physical exam was unremarkable, with normal S1S2 heart sounds and regular rate and rhythm, lungs clear to auscultation bilaterally, and benign abdominal exam. He stated he had been using cocaine for the past three days, with associated audio and visual hallucinations of “seeing and hearing death”, but was not experiencing them during the examination, with benign neurological and psychiatric assessments. Osteopathic structural examination revealed blanching viscerosomatic reflexes from T7-L2 on the right and hypertonic, asymmetric paraspinal musculature from level T6-T12, along with other somatic dysfunctions (Figure 1). Chapman’s points were appreciated on the right sixth intercostal area.

Laboratory findings revealed elevated ALT levels, with CBC, electrolytes, BUN, and creatinine within normal ranges. HCV antibody test done on previous admission was positive, however the patient declined follow-up with gastroenterology or infectious disease referrals. Subsequent EKG and cardiac enzyme levels were normal; however, he was admitted to medicine for psychiatric evaluation and referral to inpatient detox unit.

## Discussion

The patient presented here represented a complicated case of polysubstance abuse with multiple comorbidities and questionable compliance, which can vastly affect the clinical management of chronic HCV infection. While new treatments such as interferon-free and ribavirin-free oral regimens are being developed, compliance and viral load monitoring are required to ensure positive clinical outcomes [7]. Early eradication of the virus is desired as chronic infection can result in numerous complications ranging from hepatic decompensation to hepatocellular carcinoma [8]. From a public health standpoint, there is also an increased risk of transmission in untreated HCV infection, particularly in the context of IV drug use and needle-sharing [9].

HCV diagnostic testing is divided into the two broad categories of serology to detect HCV antibodies and molecular assays that detect and quantify HCV RNA [3-4]. Identifying risk factors for acute or chronic HCV infection are crucial as symptoms can be largely nonspecific and include fatigue, sleep disturbance, nausea, abdominal pain, and weight loss [2,10]. Extrahepatic diseases such as mixed cryoglobulinemia, membranoproliferative glomerulonephritis, porphyria cutanea tarda, lichen planus, and diabetes mellitus have all been associated with chronic infection; however, the prevalence is inconclusive [10]. With this ambiguity, the osteopathic physical exam can be a useful adjunctive tool in localizing pathologies in the affected organs and systems to focus on diagnostic inquiry and differential diagnosis.

**Figure 2:**
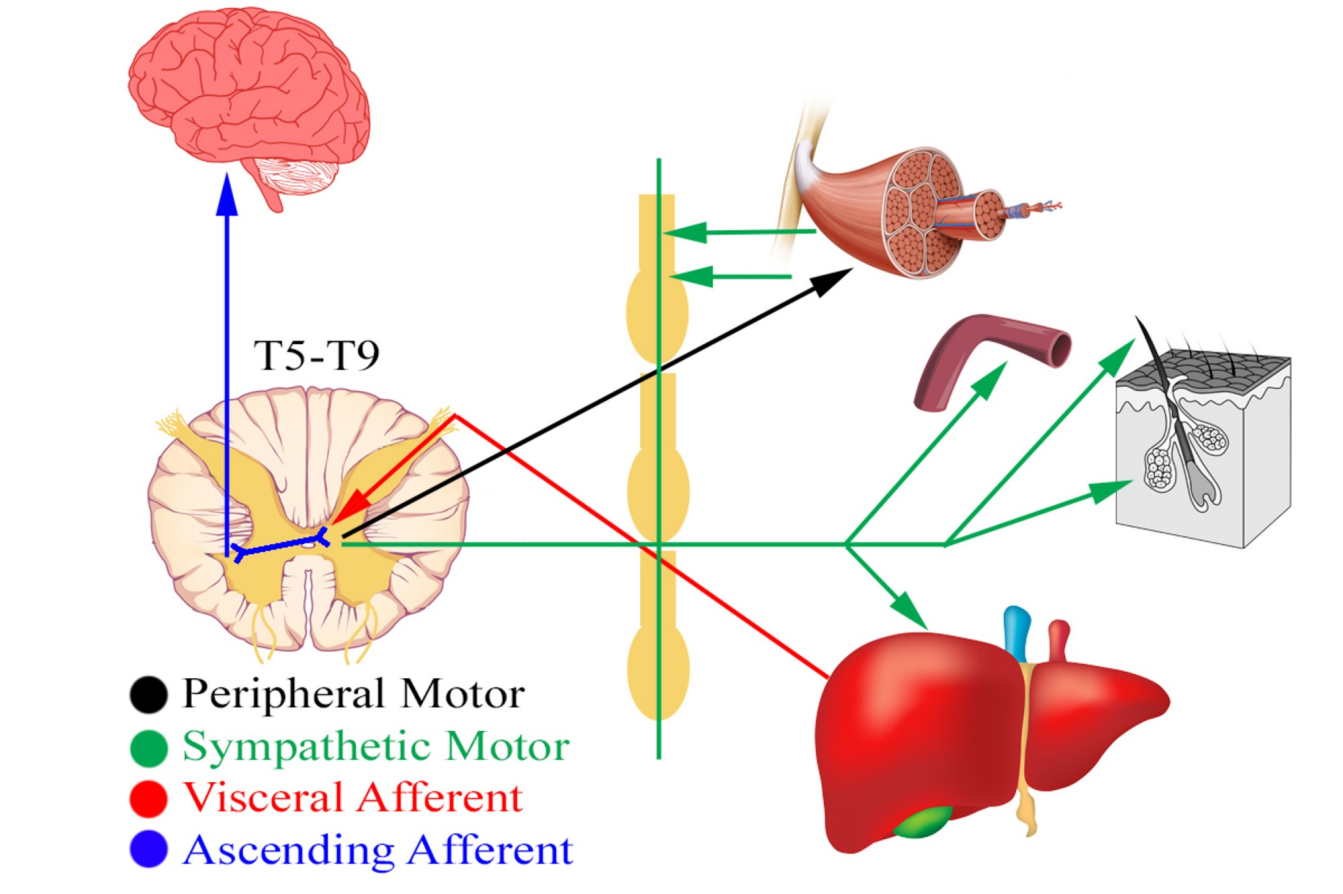
Schematic diagram of hepatic viscerosomatics and its associated musculoskeletal manifestations Original graphics created by Justin Chin

**Figure 3:**
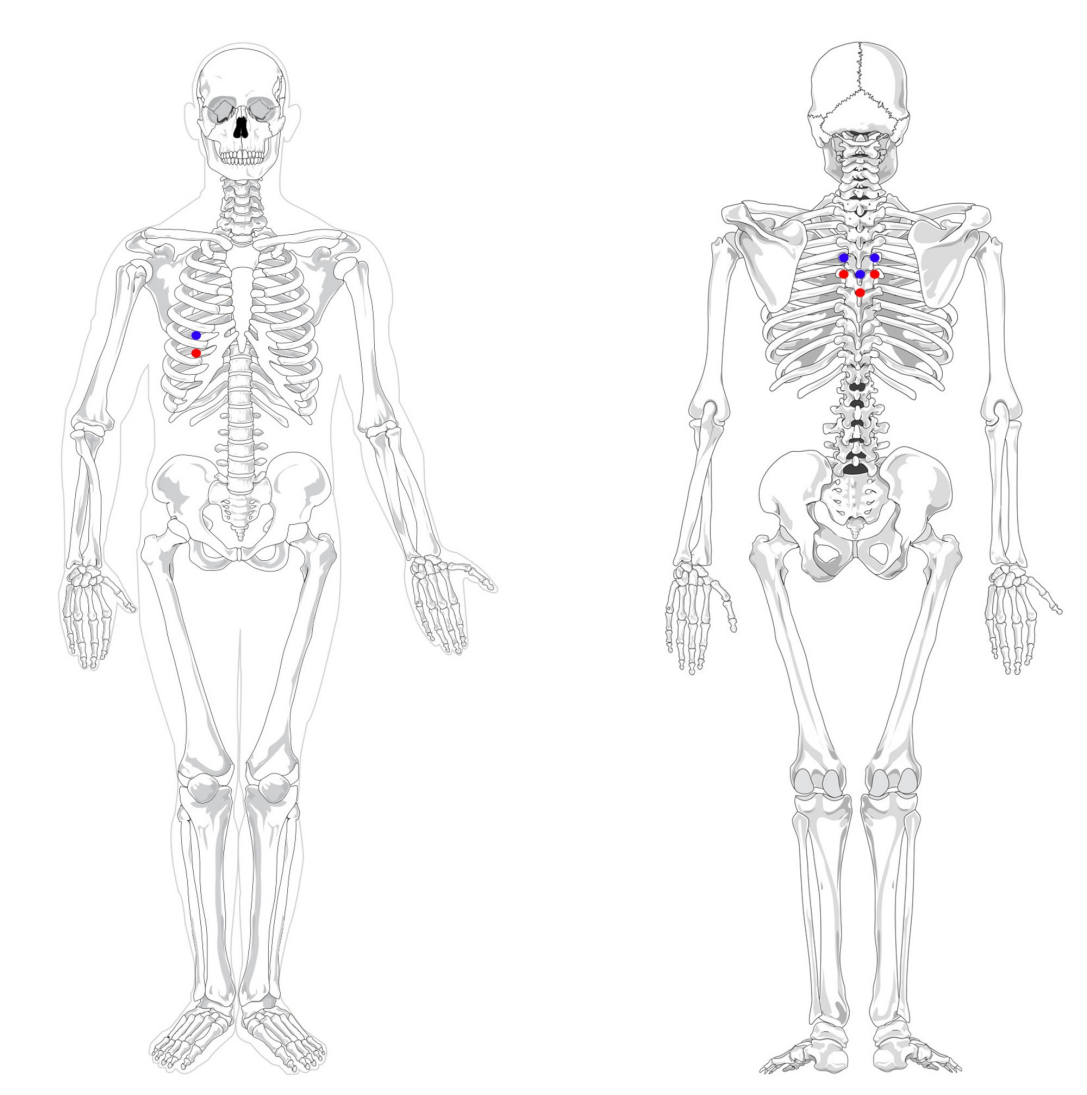
Anterior Chapman’s point locations for the liver, with its associated posterior treatment locations Blue: Chapman's point on fifth intercostal space associated with liver dysfunctions Red: Chapman's point on sixth intercostal space associated with liver and gallbladder dysfunctions Original graphics created by Justin Chin

A key component of the osteopathic physical exam is the concept of viscerosomatic reflexes, in which visceral and somatic pain afferents overlap in the dorsal horn and influence each other (Figure 2) [11-12]. This can create somatic manifestations in the thoracolumbar paraspinal region from T1-L2, which correspond to the sympathetic nervous system [13-14]. Upon palpation and visualization, these areas can present as skin erythema for acute processes versus skin blanching for chronic states [15]. Similarly, Chapman’s points are “pea-sized gangliform contractions” of musculature that associated with visceral dysfunction found elsewhere in the body (Figure 3) [15-16]. In the case of liver pathologies, patients can have an anterior Chapman’s point at the right fifth or sixth intercostal space or paraspinal muscular changes at the level of T5-T9 with correspondence to the celiac ganglion [13,15]. The presented patient had chronic skin changes on palpation, positive Chapman's points, and viscerosomatic reflexes indicating liver involvement, which lead us to the conclusion that there is a strong possibility of chronic hepatic disease originating from either HCV or substance abuse or both. Further molecular assays to quantify viral load would be logical, along with monitoring hepatic function with liver enzymes tests.

Various studies have shown the prevalence of osteopathic findings and their relation to disease pathologies [13,17-18]. For example, Chapman’s points for the lung, which are located on the third and fourth intercostal space, were found 3.9 times more in patients with pneumonia than in the general population [19]. Limited research, however, exists in the reproducibility and generalizability of these findings due to variability in operator interpretation skills and the lack of randomized, double-blinded studies [12]. Similarly, beyond the foundational studies of viscerosomatics, there has not been focused analysis of hepatic variations or pathology and the incidence of viscerosomatic findings produced on osteopathic physical exam [16,20]. Another important limitation is that these findings can mainly be used for localization as numerous organs can present similarly. For example, T5-T9 viscerosomatic reflexes can be seen with pathologies of the upper gastrointestinal tract and the Chapman’s point for gallbladder dysfunction can also be found on the right 6th intercostal space [13,15]. Nonetheless, many osteopathic physicians recognize the utility of the osteopathic physical exam as a valuable extra tool, providing additional information and developing a differential diagnosis [12,18-19].

## Conclusions

Utilizing the osteopathic physical exam, and in particular, viscerosomatic reflexes can provide additional clinical information necessary for management of disease states such as chronic hepatitis C. This additional information can assist osteopathically trained physicians in developing a differential diagnosis with the consideration of other risk factors, the chronicity of the disease process, and possible areas for osteopathic manipulative treatment in cases of referred pain. While greater research is needed to validate and establish appropriate use of osteopathic physical exam findings relative to visceral disease processes, this case presentation can be used to highlight the possibility of applying viscerosomatics to rarer diseases that lack specific laboratory studies.
